# Influence of microcrack types on macroscopic cracking of sandstone under freeze-thaw erosion

**DOI:** 10.1371/journal.pone.0328244

**Published:** 2025-08-18

**Authors:** Yanan Sun, Bing Liang, Junzu Ma, Jiaxu Jin, Daoliang Liu

**Affiliations:** 1 School of Mechanics and Engineering, Liaoning Technical University, Fuxin, China; 2 School of Civil Engineering, Liaoning Technical University, Fuxin, China; 3 Shenyang Railway Survey and Design Institute Co., Ltd, Shenyang, China; Shenyang Jianzhu University, CHINA

## Abstract

Freeze-thaw erosion is a common hazard in cold region engineering, which is capable of generating a large number of microcracks inside the rock mass. However, the effect of different forms of microcracking on the macrocracking characteristics of sandstones under freeze-thaw erosion conditions has not been elucidated. Hence, the effect of microcracking on macrocracking under freeze-thaw cycling conditions is analysed by means of a combination of acoustic emission tests and numerical simulations. The results show that the peak strength, modulus of elasticity and longitudinal wave velocity of the sandstone produced a decrease with the increasing degree of freeze-thaw erosion. When the freeze-thaw cycle reached 80 times, the ringing counts changes significantly, showing a continuous accumulation trend. The trend of b value shows that microcracking of rock samples with high degree of freeze-thaw erosion is a continuous process of accumulation. The percentage of RA and AF indicates a shift in the cracking pattern from shear to tensile as the rock specimens are subjected to an increasing number of freeze-thaw cycles. Based on a model of sandstone after freeze-thaw erosion, it is concluded that inhomogeneous variations in the displacement and force chain fields of the particles lead to different modes of fracture extension. Finally, the mechanism of the influence of along-crystal microcracking and through-crystal microcracking on the macroscopic fracture of sandstone is discussed.

## Introduction

Approximately three-quarters of China’s land area is located in cold regions, where freeze-thaw hazards pose a threat to the stability of rock masses in cold region engineering. In addition, the resource exploitation and engineering construction around the world are often influenced by freeze-thaw cycle factors [1]. Freeze–thaw erosion frequently induces deterioration in rock masses, potentially triggering severe geological hazards such as rockfalls and landslides [2]. The primary cause of freeze-thaw erosion of rock masses is the freezing and thawing of internal moisture. This phenomenon can be attributed to the expansion of ice crystals, which results in an increase in both volume and the number of microdefects within the rock [3]. This can result in a significant reduction in the load-bearing capacity of the rock mass, which presents a considerable risk to the stability of rock structures such as tunneling and coal mining operations [4]. Therefore, it is imperative to study the effect of extension derivation of micro-defects within the rock mass on macro-cracking under freeze-thaw conditions, which is of significant importance to guarantee the secure mining of rock structures and to uphold the safety of construction workers.

The various macro-physical properties of rocks are affected by freeze-thaw erosion. Existing studies have found that the expansion of ice crystals during freezing and the softening of water during thawing are the main reasons for the decrease in the mechanical strength of the rock mass. This is due to a decrease in the bond between particles, resulting in a decrease in the bearing capacity of the rock mass [5]. Furthermore, freeze-thaw erosion impacts the modulus of elasticity and poisson’s ratio of sandstone [6]. The existing studies offer an explanation that the cyclic freeze-thaw action alters the structural distribution within the particles, which consequently affects their deformation [7]. It is noteworthy that water-bearing defects in natural rocks tend to propagate into larger cracks under the influence of freeze-thaw cycles. However, these pre-existing fractures with varying geometric characteristics play a critical role in governing their failure behavior [8,9]. Consequently, an examination of microdefects within the rock is beneficial in order to clarify the impact of freeze-thaw erosion on macroscopic property alterations.

A greater number of microdefects indicates a greater degree of damage within the rock. Previous studies have suggested that many microcracks are created within the rock at high temperatures and that these microcracks eventually lead to a decrease in the mechanical properties of the rock [10]. Additionally, research has indicated that the extent of rock fragmentation subsequent to failure is correlated with the quantity of internal microcracks present prior to compression [11,12]. Furthermore, the greater the number of microcracks, the more complex the internal structure of the rock. Therefore, it can be seen that the initial number of microcracks has an influence on the formation of macroscopic cracks, as well as affecting the macroscopic properties of the rock in question. It has been proposed that the influence of initial microcracks on the stable extension phase of fracture development is considerable [13]. Nevertheless, the cracks within the rock are not discernible. Therefore, in order to gain a deeper understanding of the crack development pattern, it is essential to conduct a numerical simulation.

Finite element modelling of the freeze-thaw erosion process of rocks has been investigated, which found that the freezing period is the main stage of microcrack development [14]. And existing studies have demonstrated by means of numerical simulations that a higher number of microcracks can lead to tensile and shear damage in rocks [15]. The discrete element approach is more conducive to understanding the process of fracture formation, as it responds to the interactions between the particles [16]. In the previous studies, it was demonstrated that the crack extension paths were deflected by the freeze-thaw cycling effect using the discrete element approach [17,18]. It is important to note that the results derived from numerical calculations represent specific patterns and should be verified through experimental means.

There are a number of techniques that can be employed to observe and collect microcracks, including scanning electron microscopy (SEM), magnetic imaging, and CT scanning [19,20]. Nevertheless, these techniques are only able to ascertain the present crack morphology and do not offer a reliable indication of whether the damage is tensile or shear in nature. Acoustic emission is widely used in rock as a technique to detect cracks [21,22]. Parameters such as ring count, duration, and amplitude can be captured by the acoustic emission device, through which the distribution of spatial cracks within the sandstone as well as the nature of the cracks can be recognised [23]. It has been demonstrated that the sample entropy of the acoustic emission signals generated by tensile and shear cracks exhibits significant divergence. Consequently, the nature of the crack can be discerned through this method [24]. Subsequently, it was discovered by such methods that a large number of tensile cracks were produced in rocks after freeze-thaw erosion [25,26]. However, the influence of the nature of cracking within the rock under freeze-thaw cycling conditions on the macroscopic cracking mechanism is not well elucidated.

This study tends to elucidate the effect of microcracks on the macroscopic cracking of sandstone under the action of freeze-thaw cycles from the perspective of the nature of microcracks. Firstly, uniaxial compression and acoustic emission experiments were carried out on sandstones with five numbers of freeze-thaw cycles to test the changing rules of macroscopic physical properties and acoustic emission signal characteristics of sandstones under freeze-thaw erosion. Then, the whole test process was simulated with the aid of the discrete element method, and the variation rules of the displacement field and force chain field of sandstone particles under the freezing and thawing effect were obtained, and the rupture morphology of the specimens in the test and simulation was compared. Finally, the effect of different types of cracking under freeze-thaw conditions on the macroscopic cracking characteristics of sandstones is discussed.

## Materials and methods

### Preparation of rock specimens

In this study, the rock specimen used belongs to fine-grained dense sandstone, which comes from Zigong City, Sichuan Province. According to the national standard DZ/T 0276.18–2015, in this study, the rock samples are processed into standard cylindrical specimens with a diameter of 50 mm and a height of 100 mm. It is required that the non-parallelism of the two end surfaces should not exceed ±0.05 mm, and the deviation between the end surfaces and the axis should be perpendicular to each other, and the deviation should not exceed ±0.25°. The physical and mechanical properties of rock specimens used in this study are presented in Table 1.

**Table 1:** Physical and mechanical properties of rock specimens.

Density/g·cm^-3^	Elastic Modulus/GPa	Uniaxial Compressive Strength/MPa	Longitudinal Wave Velocity/(km·s^-1^)	Porosity/%
2.32	3.75	30.82	2.357	10.37

### Mechanical and acoustic emission tests

Uniaxial compression tests are carried out on prepared standard rock specimens. The instrument used for the uniaxial compression test is the TAW-2000 triaxial test press. During compression, the cracking signal is acquired by means of acoustic emission tests. A four-channel acquisition method is employed, whereby four probes are attached to the left and right sides of the specimen. The subsequent setting of the threshold is designed to ensure the accurate capture of the cracking signal. To avoid interference from surrounding noise, the acoustic emission collection threshold was set at 40 dB.

From the waveforms obtained in the acoustic emission test, it is possible to ascertain the evolution pattern of the characteristic parameters, including energy, ringing counts, rise time and duration, when the specimen is subjected to pressure.

### Discrete element theory and model building

#### Particle flow methods.

The initial conceptualisation of cohesive particles is posited by Potyondy et al. within the context of the discrete element method (DEM) theory [27]. This model has subsequently been employed extensively within the domains of material science and environmental engineering.

**Fig 1:**
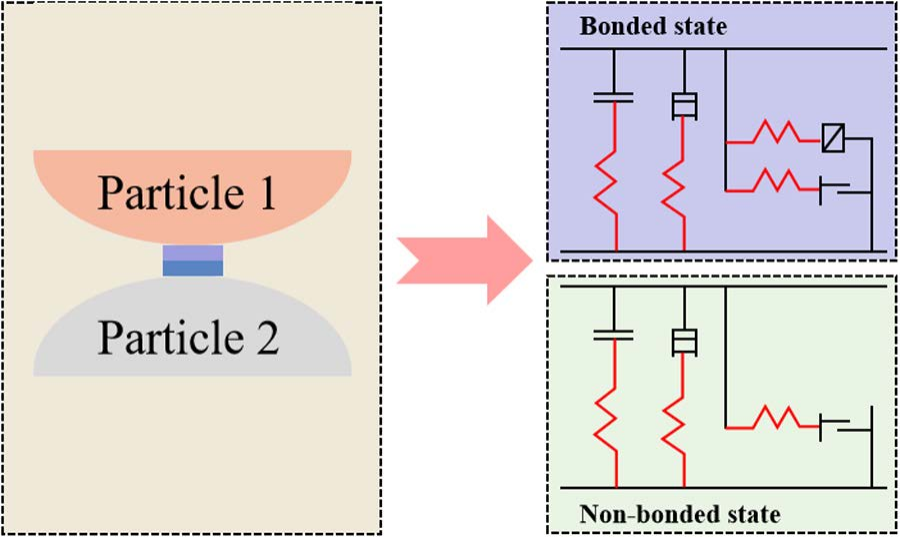
Parallel bond model.

The parallel bonding model (PBM) is a type of bonded particle model that is thought to be effective in replicating the mechanical behaviour of rock materials. Fig 1 illustrates the fundamental principle of the PBM. When the particles are in a bonded state, the linear and parallel bonding elements are activated, thereby enabling them to collectively bear the load. The elimination of the bonded state between the particles results in the linear element within the particles becoming the sole functional component, which is indicative of a state of destruction.

#### Freeze-thaw cycle modelling.

In order to model the freeze-thaw cycle on sandstone, three preliminary assumptions must be established. Firstly, the sandstone model is simplified into two parts: mineral particles and water molecules. Secondly, the penetration between mineral particles and water molecules is not considered. Finally, the transfer of temperature through the water molecules is uniform. The change in volume of water molecules in the pores during freeze-thaw can be expressed as:


$$V_{0}=\frac{4}{3}\pi r_{0}^{3}$$ (1)

$$u=r_{0}\frac{p}{E}\frac{\left[1+v+2(1-2v)n\right]}{2(1-n)}$$ (2)

$$p=\frac{0.029}{\frac{1}{E}\frac{1+2n+(1-4n)v}{2(1-n)}+1.029\frac{1-2v_{i}}{E_{i}}}$$ (3)

$$V_{E}=\frac{4}{3}\pi (r_{0}+u{)}^{3}=V_{0}+\Delta V$$ (4)

where $V_{E}$ denotes the volume of water molecules after freezing, $V_{0}$ denotes the volume of water when unfrozen, ΔV is the volume increment of water after freezing, $r_{0}$ is the initial radius of the pore, u is the expansion of the pore radius, *p* is the ice crystal pressure, *n* denotes the porosity of the rock matrix, *E* and *µ* are the modulus of elasticity and poisson’s ratio of the rock matrix, respectively and $E_{i}$ and $v_{i}$ are the modulus of elasticity and Poisson’s ratio of the ice crystals respectively.

Therefore, the volume change function of the water molecule is:


$$V=\{\;\;\;\begin{array}{cc} {}*20lV_{0}+\Delta V(1-w_{u}) & \;T\leq 0^{\circ }C\\ V_{0} & \;T>0^{\circ }C \end{array}$$ (5)

$$w_{u}=1-[1+0.139(\frac{1}{T}{)}^{1/3}\ln(\frac{1+e^{0.268T}}{2})](1-e^{0.268T})$$ (6)

Where $w_{u}$ is the water content of unfrozen water.

The number of freeze-thaw cycles is controlled by calling the cosine function as in Eq. (7):


$$T=20\cos(\frac{\pi t}{30})$$ (7)

where *T* denotes the target temperature and *t* denotes time.

**Fig 2:**
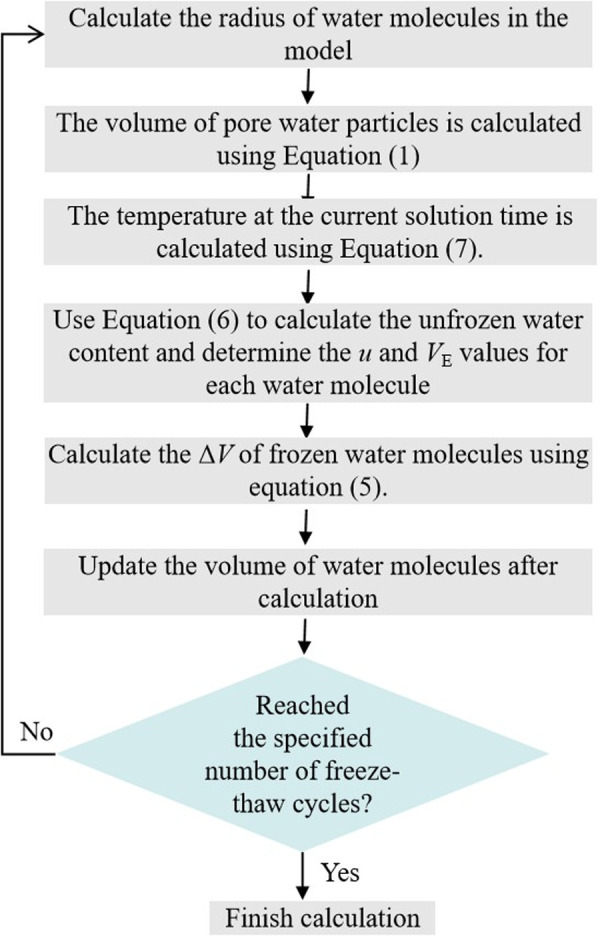
The simulated freeze-thaw cycle process.

The above equation delineates the phase transition behavior of water molecules confined within a single pore during the freeze-thaw cycle. The calculation process is illustrated in Fig 2.

#### Rock matrix model.

**Fig 3:**
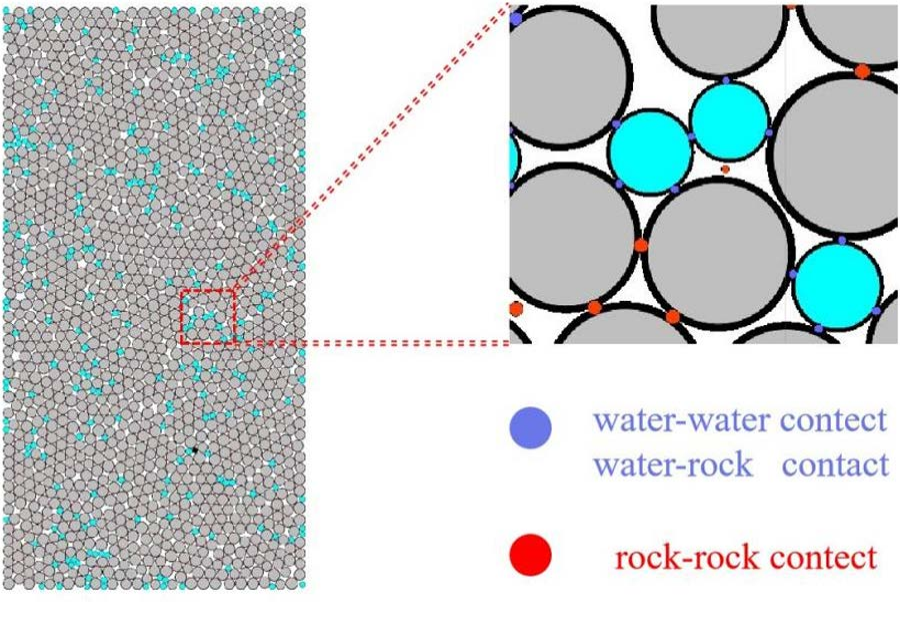
Model of sandstone under the action of freeze-thaw erosion.

Macroscopic mechanical properties and cracking characteristics of sandstone under freeze-thaw erosion simulated by PFC^2D^. The model is constructed according to the dimensions of the actual specimen employed in the test, which is a standard 50 mm x 100 mm cylinder. As it is two-dimensional, it is represented as a rectangle, as shown in Fig 3.

## Results and discussion

### Evolution of macroscopic properties of freeze-thawed sandstone

#### Macro-mechanical property.

**Fig 4:**
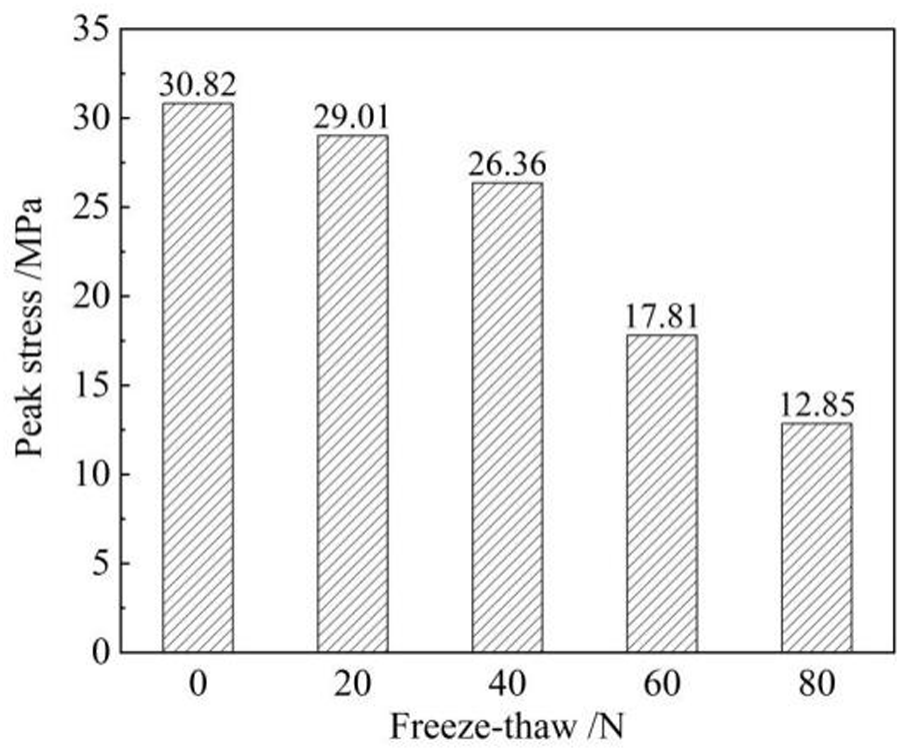
Effect of freeze-thaw cycles on the peak strength of sandstones.

Analysing variations in the peak strength of sandstones provides a good understanding of the negative effects of freeze-thaw erosion. As shown in Fig 4, the peak intensities decrease by 5.9%, 14.5%, 42.2%, and 58.3% when the number of freeze-thaw cycles is increased from 0 to 80 times, respectively. It is worth noting that the peak stress decreases significantly when the number of freeze-thaw cycles exceeds 40. This suggests that cumulative erosion by freezing and thawing can significantly alter the mechanical properties of sandstones, which is consistent with previous studies [28]. In addition, variations in the macroscopic strength of sandstones are essentially due to the continuous accumulation of internal micro-defects. The present study found that the internal pores gradually became dominated by connected pores with repeated frost heaves [29]. And the microfractures formed after the evolution of the connecting holes are relevant to the fracture mode of the sandstone after the destabilised state [30]. This will continue to be discussed in depth in subsequent chapters.

#### Longitudinal wave velocity.

**Fig 5:**
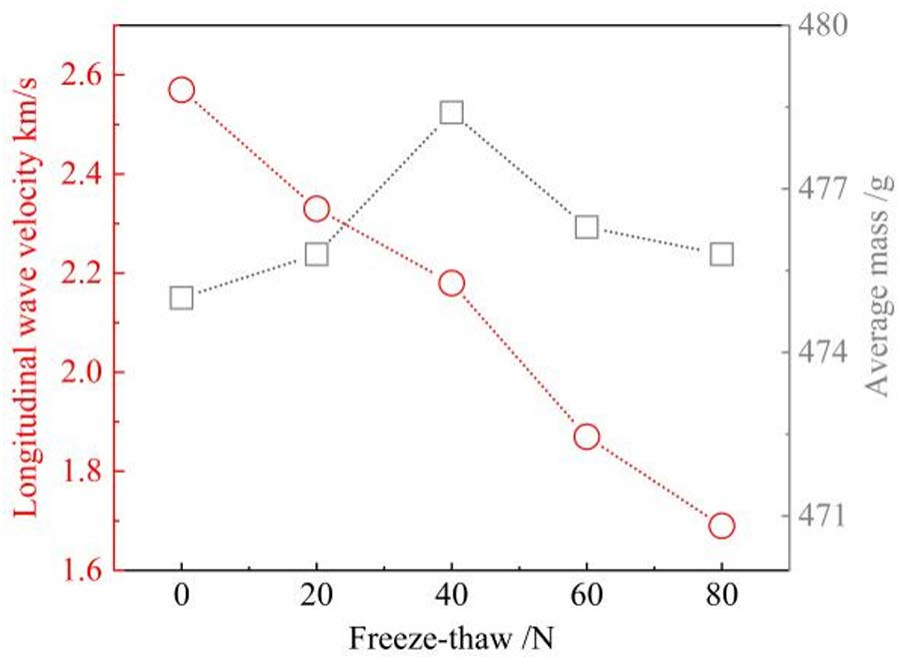
The evolution pattern of longitudinal wave velocity and average mass.

A non-metallic ultrasonic monitor can be used for non-destructive monitoring of the interior of sandstone. By collecting the longitudinal wave velocity of the rock samples, the evolution of internal micro-defects in the specimens can be preliminarily assessed [31]. As shown in Fig 5, as the number of freeze-thaw cycles increases, the longitudinal wave velocity shows a decreasing trend. This is due to the internal micro-defects causing the incident wave velocity to attenuate during reflection. It indicates that a high number of freeze-thaw cycles results in the formation of numerous micro-defects within the sandstone, which is consistent with previous studies [32]. Additionally, the average mass of the sandstone increases initially before declining, which indicates that after the formation of new microdefects, water molecules continue to fill these defects under capillary action. This is the reason for the initial increase in sample mass during the early stages of freeze-thaw cycles. As water softens and freeze-thaw forces erode [33], the reduction in cementation leads to particle detachment, thereby reducing the overall mass of the sandstone.

#### Elastic modulus and poisson’s ratio.

The macroscopic properties of sandstones can be altered by the development of internal microdefects following freeze-thaw damage [34]. Furthermore, when sandstone is subjected to external loading, changes in the internal structure induce the development of uncoordinated deformation of the rock mass in space [35]. Therefore, the relationship between the number of freeze-thaw cycles and the elastic modulus and Poisson’s ratio is analysed [36].

**Fig 6:**
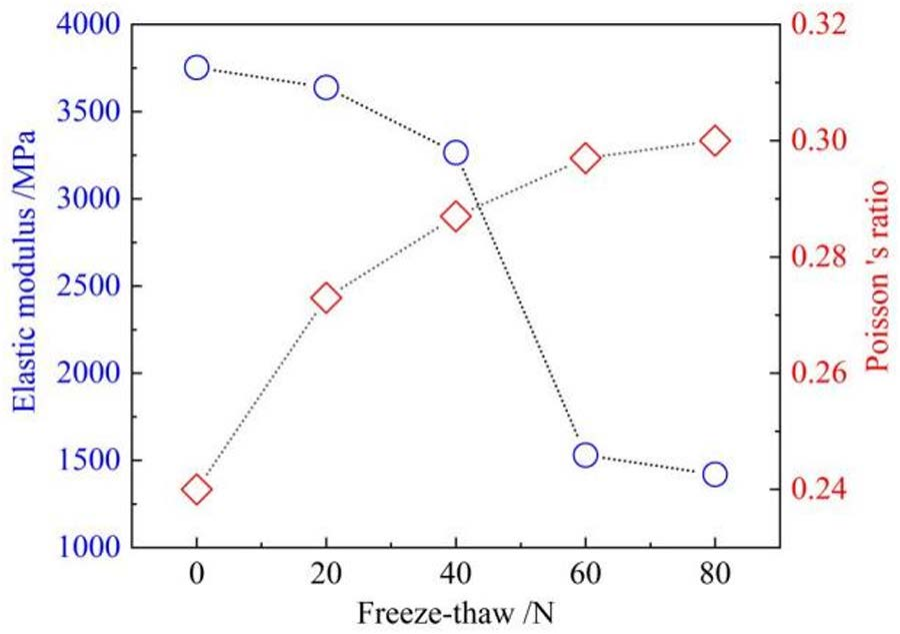
Relationship between the number of freeze-thaw cycles and the elastic modulus and poisson’s ratio.

As illustrated in Fig 6, the elastic modulus of sandstone exhibits a gradual decline, while the Poisson’s ratio gradually increases with the accumulation of freeze-thaw cycles. Previous studies have shown that the evolution of microdefects can have an impact on the trend of the elastic phase in the stress-strain curve [37]. The higher the number of freeze-thaw cycles, the greater the deformation of the sandstone at the same stress level. This suggests that the sandstone is progressively less brittle and that freeze-thaw erosion has exacerbated the ductile deformation. In addition, the deformation of sandstone includes transverse and longitudinal deformation [38]. From the change rule of Poisson’s ratio, it can be found that the higher the number of freeze-thaw cycles, the greater the transverse deformation of sandstone specimens. It has been found that the deformation of a rock under pressure may be related to the type of damage it will undergo [39]. Therefore, it is necessary to study the cracking characteristics of sandstone.

### Acoustic emission analysis

#### Ringing count.

**Fig 7:**
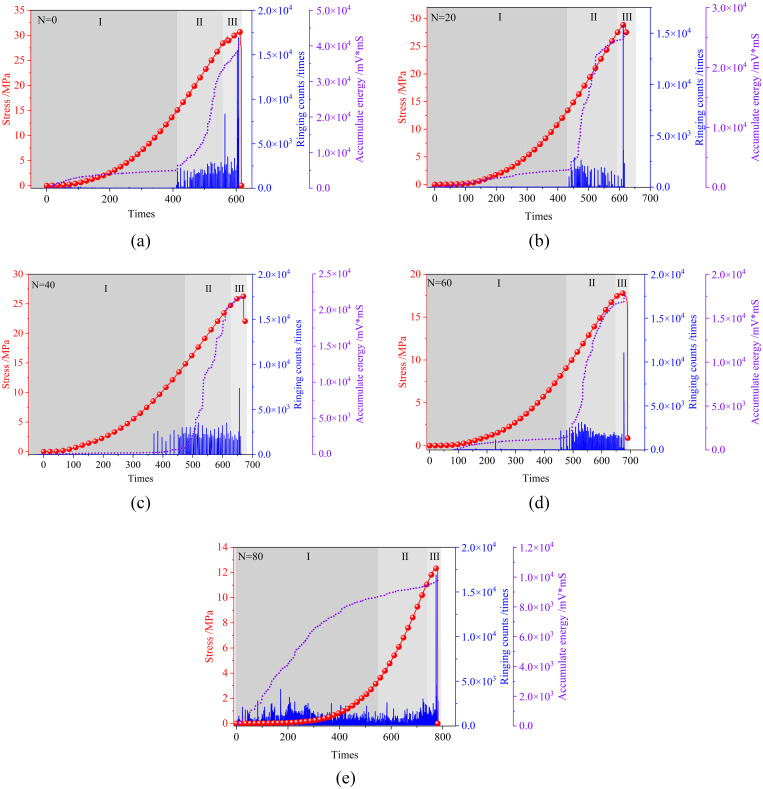
Acoustic emission signal and energy evolution characteristics after freeze-thaw cycles in sandstones (a, b, c, d, and e represent N = 0, 20, 40, 60, and 80, respectively.).

When sandstone is fractured under pressure, the rapid release of energy generates elastic waves, and the sensor converts the vibrations into electrical signals, thus enabling the capture of rock cracking features [40]. As illustrated in Fig 7, the variation characteristics of the ringing counts for the first 60 freeze-thaw cycles specimens are essentially identical. The compaction phase basically ends around 400s and cracking signals of lower amplitude begin to appear within the sandstone. Finally, a high-frequency prominent signal appears, characterising the complete destruction of the sandstone specimen. However, the specimens under 80 freeze-thaw cycles have shown small cracking signals at the beginning of compression, until the final damage produced a sudden change in the signal. This phenomenon suggests that when freeze-thaw erosion reaches a certain level, micro-cracking within the sandstone is affected in time. Existing studies have found that the generation of microcracks after erosion also has an effect on the final fracture morphology of sandstone specimens [41]. Changing the pattern of rock cracking is not only caused by the presence of frost heaving pressure but is also related to the softening of crystals by water [42]. This continues to be discussed in subsequent chapters.

Additionally, the trend in the evolution of cumulative energy shows a significant inflection point for the first 60 freeze-thaw cycles, which indicates that a large amount of micro-cracking occurs at this time. Nevertheless, the cumulative energy of the sandstone specimens is gradually increasing after 80 freeze-thaw cycles, which indicates that the cracking behaviour is accompanied by the whole process of rock compression. Therefore, specimens with a low degree of freeze-thaw erosion crack after compaction.

#### b value.

Acoustic emission b-value patterns contribute to a better understanding of rock cracking mechanisms. The principle of least squares is used to calculate the ratio of low-amplitude events to high-amplitude events, i.e., the b-value [43]. Hence, the investigation of the evolution pattern of b-value is more conducive to the analysis of the effect of freeze-thaw cycling on the cracking characteristics of sandstones.

**Fig 8:**
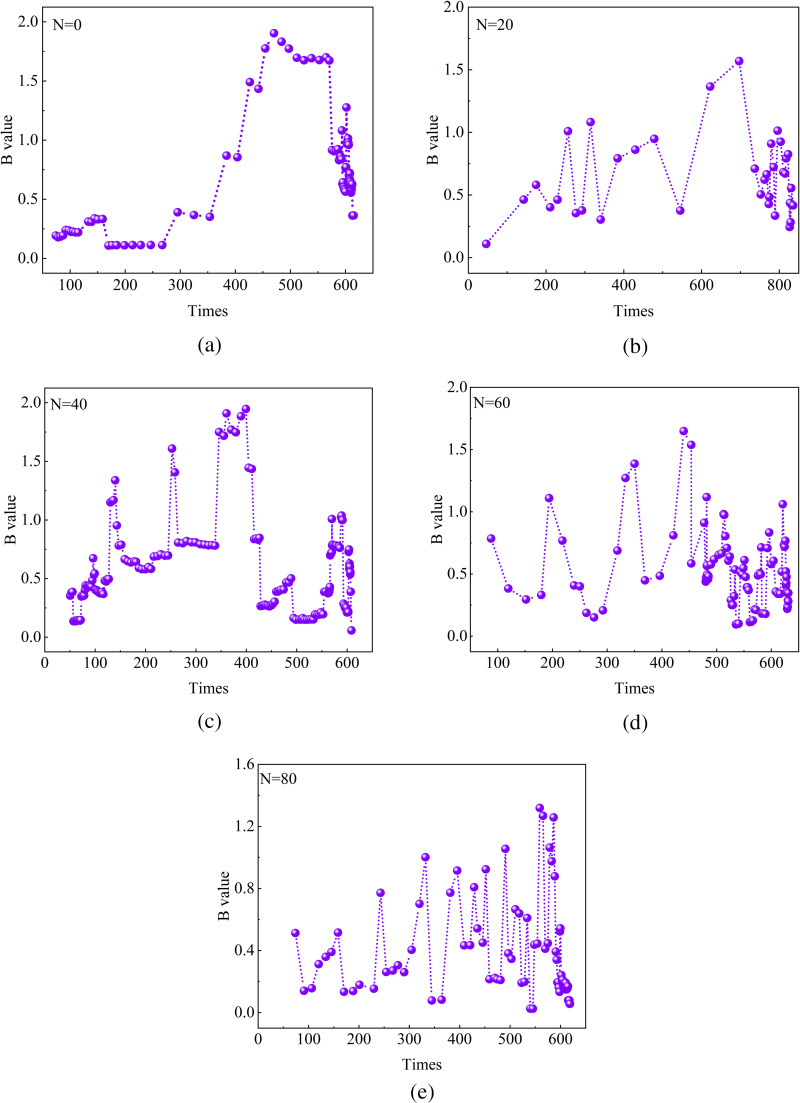
Characterization of acoustic emission B-value evolution (a, b, c, d, and e represent N = 0, 20, 40, 60, and 80, respectively.).

Firstly, the trend of the b-value of the sandstone specimens shows that it is increasing and then decreasing. However, the frequency of b value variations is low during the first 40 freeze-thaw cycles, as shown in Fig 8(a),(b) and (c). Then the trend of the b-value curve shows that the number of curve fluctuations gradually increases with the increase of the number of freeze-thaw cycles. The observation of continuous fluctuations in b-values at high freeze-thaw cycles indicates that microcracks continue to accumulate and penetrate at this stage of the process, as shown in Fig 8(d) and (e). During this stage, the sustained development of microcracks produces low-amplitude acoustic signals, resulting in an elevated b-value. The observed decline in b-values across all specimens indicates that the formation of macrocracks as a result of the convergence of microcracks ultimately leads to the destruction of the sandstone. The generation, convergence and penetration of microcracks have been demonstrated by previous studies [44]. Macrocrack formation releases substantial energy, resulting in the generation of high-amplitude signals. As the proportion of such signals increases, the b-value correspondingly decreases. However, existing studies have shown that cracking is a complex process that involves shearing, stretching, etc [45]. Therefore, understanding the dominant damage modes of freeze-thawed sandstones is better facilitated by analyzing how microcracks crack.

#### RA-AF value.

In existing studies, it is possible to analyze the destructive mechanisms of material cracks by linking two parameters, the average frequency (AF) and the rise time/amplitude (RA) [46]. Tension cracks exhibite high AF and low RA distribution characteristics, shear cracks exhibits the opposite trend [47]. Consequently, the cracking pattern of fractures under freeze-thaw erosion can be fully understood by analyzing the characteristics of AF and RA.

**Fig 9:**
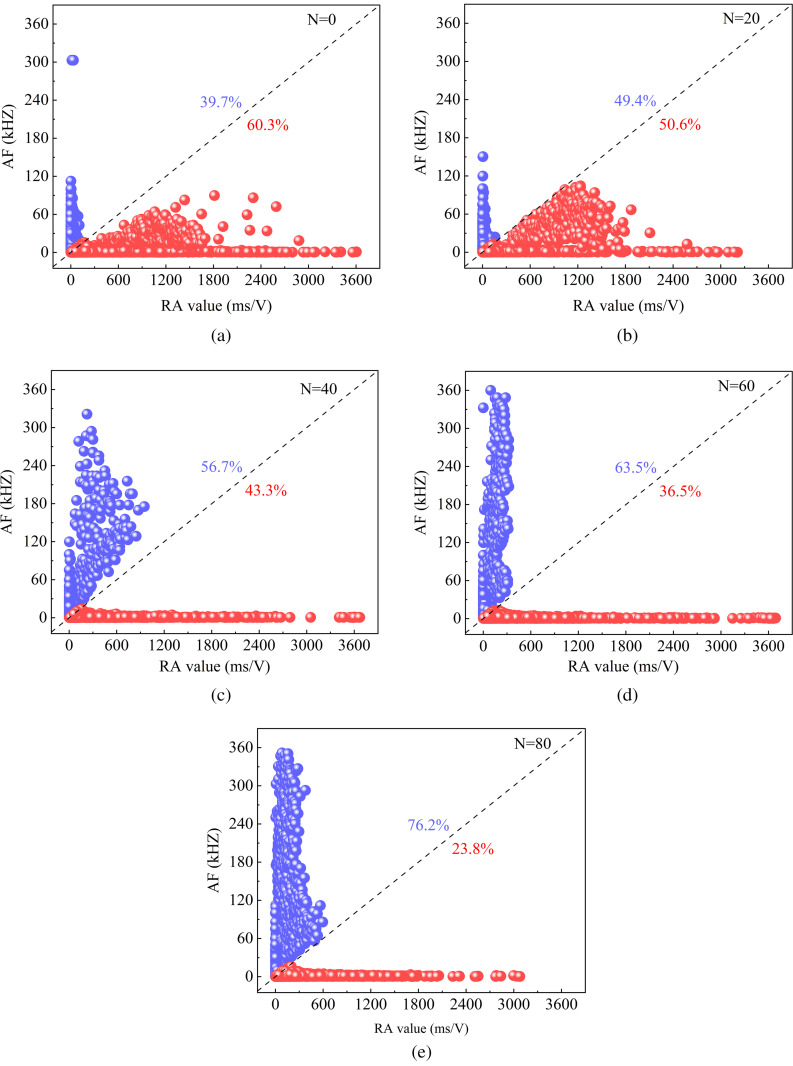
Evolutionary characteristics of RA and AF in sandstone under different numbers of freeze-thaw cycles. (a, b, c, d, and e represent N = 0, 20, 40, 60, and 80, respectively.).

As shown in Fig 9(a) and (b), the acoustic emission cracking of sandstone during the first 20 freeze-thaw cycles is characterized by high RA and low AF. The frequency of high RA decreases with increasing freeze-thaw cycles from 20 to 80. It is noteworthy that the distribution of RA gradually moves closer to the dashed line when the number of freeze-thaw cycles increases from 0 to 20, which indicates that the signal of shear cracks generated within the sandstone gradually weakens. When the acoustic emission signal AF is high, the distribution of AF gradually moves away from the dashed region as the number of freeze-thaw cycles increases from 40 to 60, which indicates a gradual transition to tensile cracking within the sandstone. Present studies have found that frost heave pressure from water-ice phase transition is the main cause of induced cracking of internal fractures in rocks [48]. In addition, sandstone cracking patterns are related to the direction of fracture derivation and extension patterns [49]. When the sandstone reaches 80 freeze-thaw cycles, the area where AF occurs gradually approaches near the dotted line, which further proves that the damage mode of sandstone due to multiple freeze-thaw erosion is a mixed cracking mode dominated by tensile cracking. This is due to the random derivation and extension of fractures, which is consistent with existing studies [50]. Thus, in this study, it was found that the cracking pattern of sandstone shifted from shear to tensile cracking as the number of freeze-thaw cycles increased, and that a mixed pattern of cracking appeared within the specimen at high freeze-thaw cycles.

### Validation of the model

#### Calibration of model parameters.

**Fig 10:**
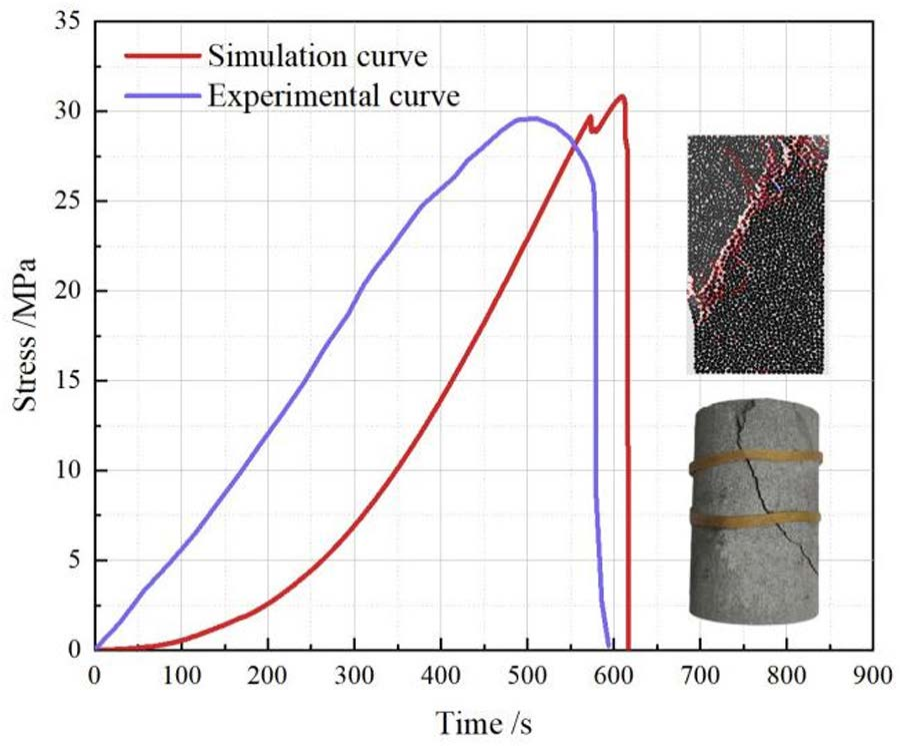
Comparison of stress-strain curves and damage patterns.

Table 2 demonstrates the specific meso-parameters of the cylindrical specimen models, which are calibrated by comparing the difference between the uniaxial compressive strength of the model and that of the intact rock sample to determine the magnitude of specific meso-parameters. The specific comparison results are shown in Fig 10. Due to the difficulty in fully simulating natural defects within the rock in the modeling of the rock specimens, the curves vary in their trends during the compaction phase. The experimentally measured compressive strength was 30.82 MPa and the modulus of elasticity was 3.75 GPa. The PFC^2D^ simulation yielded a uniaxial compressive strength of 29.62 MPa and a modulus of elasticity of 3.62 GPa, and the numerical results better reflect the fracture pattern and peak strength of the sandstone. Therefore, it can be determined that the fine view parameters of the cylindrical model are set reasonably.

**Table 2:** Meso-parameters of numerical simulation.

Contact type	Particle contact modulus *E*_c_/GPa	Particle stiffness ratio	friction coefficient µ	Parallel bonding modulus $\begin{array}{c} \overset{―}{E_{c}} \end{array}$ /GPa	Parallel bond normal strength/MPa	Parallel bond tangential strength/MPa
Rock-Rock	1.7	3.1	0.5	2.3	5.6	5.3
Water-Water	1.7	0.5	0.5	2.3	2300	1300
Rock-Water	1.7	0.5	0.5	2.3	2300	1300

### Evolution of loaded rupture

Analyzing the cracking inside the specimen using the discrete element method gives a clearer understanding of the forces between the internal micrometric particles as well as the relative displacements. Existing studies have found that rock cracking is divided into four stages: a slow development stage, a smooth development stage, and an accelerated derivation stage, respectively [45].

**Fig 11:**
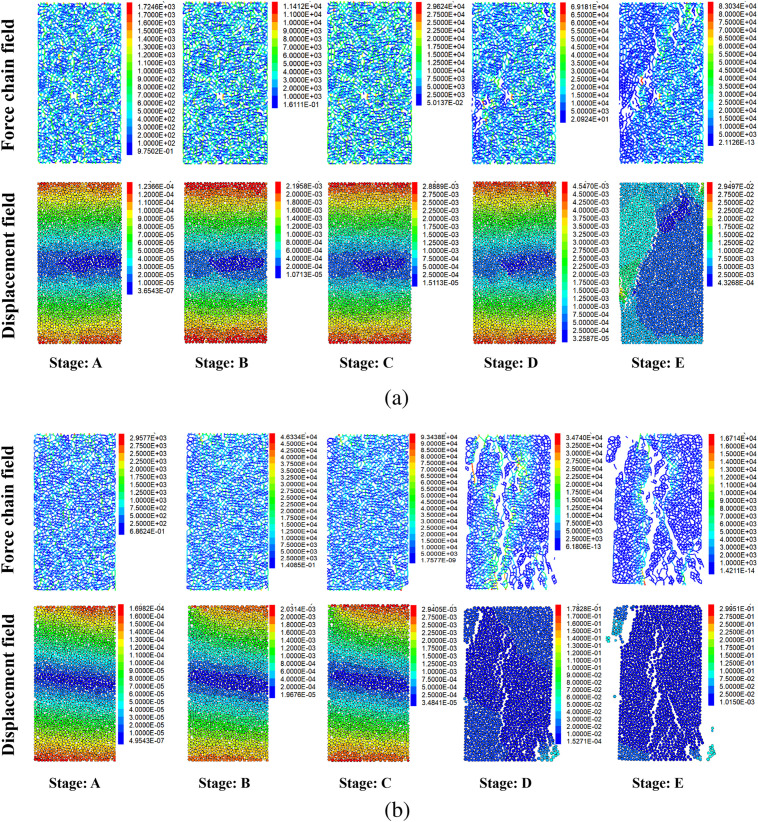
Evolution of specimen cracking under pressure. (a, b represent 0 and 80 freeze-thaw cycles, respectively.).

Fig 11 depicts the evolution of the force chain field as well as the displacement field during the rupture of the specimen under 0 and 80 freeze-thaw cycles. During the early stages of compression, without freeze-thaw cycles, the distribution of the force chain in the specimen is uniform. And the conduction of the force chain always occurs from particle to particle. The creation of microcracks can interrupt the continuity of the force chain structure. Therefore, it can only continue along the perimeter of the microcrack. However, microporosity exists naturally in rocks. The contact between particles during compression often results in shear slip due to the presence of microdefects. At this point, the specimen needs to overcome the sliding friction effect between the particles. This means that in the absence of erosion, the interaction between the particles is dominated by the shear effect. At high freeze-thaw cycles, tensile rupture surfaces tend to form due to the reduced adhesion between particles. As a result, the direction of conduction of the force chain is predominantly transverse, which is the main reason for the higher number of tensile cracks. In contrast to the 0 freeze-thaw cycle specimens, after 80 freeze-thaw cycles, the specimens showed a more pronounced accelerated stage of crack evolution. This is not only related to a decrease in adhesion, but is more likely to be caused by the rupture of the micrometric particles [51].

It is worth noting that freeze-thaw cycles have a significant effect on the variation of the displacement field. During the first four compression stages, the displacement field of the specimen with 0 freeze-thaw cycles varies uniformly. However, the variation of the displacement field of the specimens with 80 freeze-thaw cycles is non-uniform. This is due to the random distribution of micro-defects caused by freeze-thaw erosion, which changes the travelling path of the particles during compression, thus affecting the final damage pattern [52]. And a significant tilt of the displacement field can also be detected. This is due to a decrease in particle rigidity, which is consistent with previous study [53]. From the final damage pattern of the specimen, the change laws of the displacement field are also an important factor affecting the cracking of the specimen.

### Comparison of macro-destructive patterns

**Fig 12:**
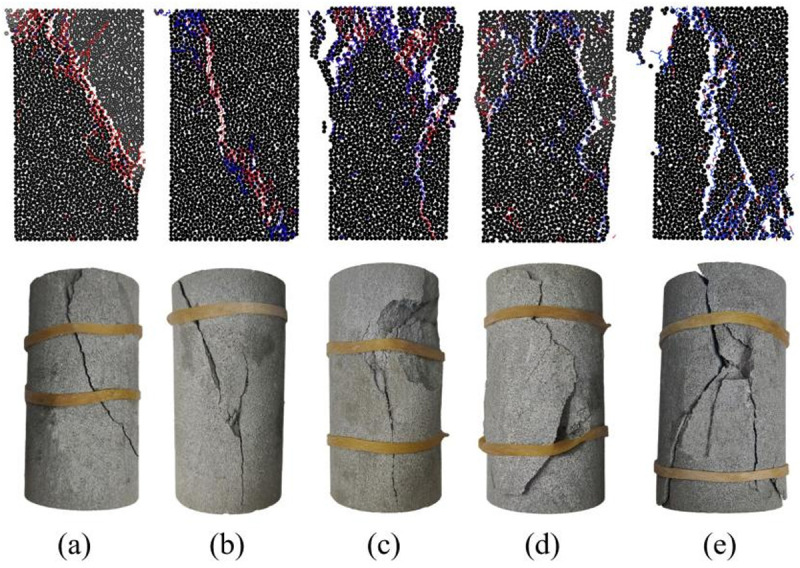
Characteristics of crack evolution in sandstones under different numbers of freeze-thaw cycles. ((a, b, c, d, and e represent N = 0, 20, 40, 60, and 80, respectively.).

Fig 12 illustrates the macroscopic damage patterns of sandstone specimens under five numbers of freeze-thaw cycles. It can be noticed that the damage occurring in the specimen is mainly dominated by diagonal shear cracking during the first 20 freeze-thaw cycles. Derivative cracks accompanied by the main crack extension are found in the N = 20 specimen. This is due to the fact that sandstone in lower freeze-thaw erosion destruction has to overcome the bonding between particles as well as friction, and the bonding force overcome is radial when compressed in the longitudinal direction [54]. The anisotropy of the particle distribution results in the particles being in contact with each other in a staggered form. Thus it is easy to form cracked areas along the shear direction. In the existing studies, it is discovered that the breaking surface of the rock is always along the direction of the maximum principal stress [55]. It indicates that the direction of maximum principal stress is at an angle to the central axis for the less eroded specimens. As the degree of freeze-thaw erosion increases, the cracks appearing in the specimen are mostly in the axial direction, as shown in Fig 12(c) and (d). The cracking mode at this point is tensile cracking, which is consistent with previous research [56]. It is noteworthy that many flaky derivative cracks distribute around the main cracks after 80 freeze-thaw cycles, as shown in Fig 12(e). At this point, tensile and shear cracks exist simultaneously, and this cracking mode is classified as mixed cracking. This phenomenon is related to the complex stress state inside the specimen [57].

### Quantitative and qualitative analysis of cracks

**Fig 13:**
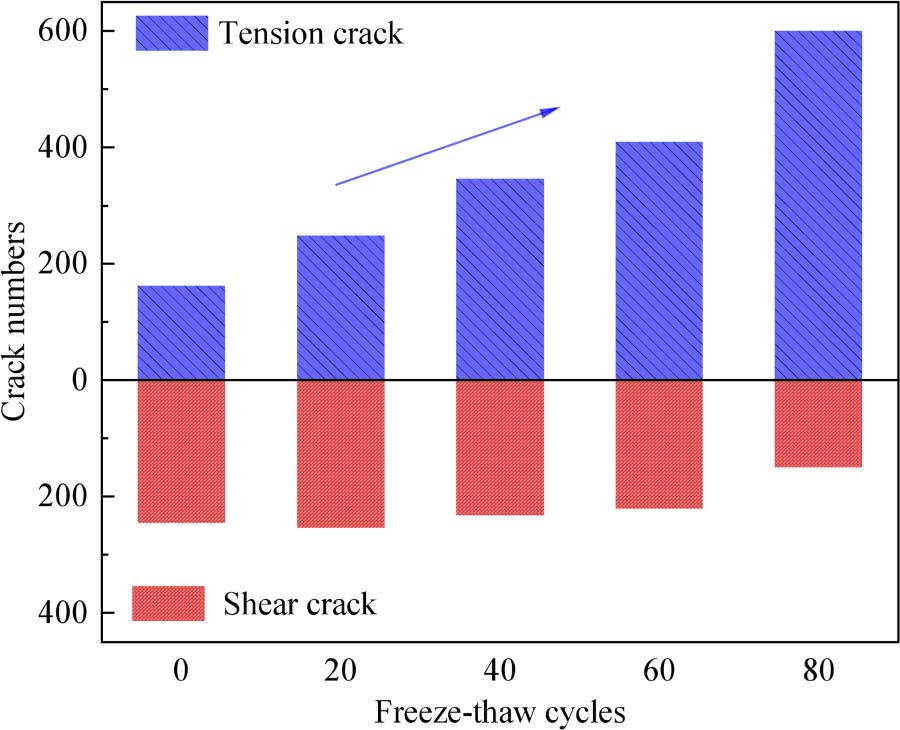
Variation of the number and type of cracks with the number of freeze-thaw cycles.

The number and type of cracks after cracking of the simulated specimens are calculated, as shown in Fig 13. The number of tensile cracks is positively correlated with the number of freeze-thaw cycles, and shear cracks exhibit a decreasing trend. Furthermore, when the number of freeze-thaw cycles is increased to 20, the number of tensile cracks gradually equalises to the number of shear cracks. When the freeze-thaw erosion gradually increased, tensile cracks accounted for significantly more than shear cracks. This phenomenon is consistent with subsection 3.2.3. Previous studies have found that the expansion of ice crystals reduces the bonding between rock particles, allowing internal microdefects to deform plastically [58]. These microdefects induce tensile damage to the rock. Additionally, at the macroscopic level, the main difference between tensile and shear cracks is the angle between the crack surface and the direction of the principal axis. It has been demonstrated that the angle of cracks can approach the axial direction as the number of freeze-thaw cycles increases. Furthermore, it has been established that freeze-thaw erosion diminishes the shear capacity of rocks [59]. However, other scholars have tested sandstone through the Brazilian Splitting Experiment, which found that after freeze-thaw cycles, the dynamic tensile strength of sandstone decreases as well [60]. Thus, this confirms that the final damage mode of sandstones with a high degree of freeze-thaw erosion is mixed cracking damage.

## Discussion

The effects of freeze-thaw erosion on sandstones show the derivation of internal micro-defects leading to a decrease in mechanical strength, longitudinal wave velocity and different types of macro-cracking patterns. In a sense, large macroscopic cracks are the result of the convergence of many tiny cracks. Therefore, the discussion section begins by elucidating the effect of freeze-thaw erosion on the mode of microscopic crack derivation in sandstones. Subsequently, the effect of the presence of microcracks on the macroscopic cracking pattern of sandstones is discussed. Finally, the limitations of the theoretical model are discussed.

### Effect of freeze-thaw cycles on microcracking characteristics

**Fig 14:**
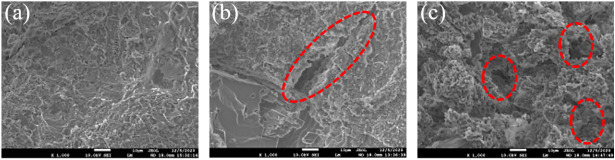
Microstructure within the sandstone. (a, b, c represent 0, 40 and 80 freeze-thaw cycles, respectively.).

Previous studies have extensively investigated the environmental factor of freeze-thaw cycles [5,61]. The main cause of freeze-thaw erosion is due to the weakening of adhesion and the expansion of the pores by ice crystals. From a microstructural point of view, there is indeed a behaviour of gradual connectivity of the pores within the sandstone, as shown in Fig 14. As the number and volume of connected pores continue to increase, microdefects dominated by freeze-thaw erosion appear within the sandstone. However, at this point these microdefects are almost always extended along the edges of the inter-particle contact. These types of cracks are collectively referred to as along-crystal cracks [62]. Therefore, the direction of extension of the cracks along the crystal depends only on the direction of the crack ends. Furthermore, It has also been shown that the softening of the water reduces the stiffness of the particles, which can lead to cracking when microcracking occurs directly through the crystal [63]. This type of crack is known as a through-crystal crack [64]. The formation of perforating cracks is more random. It is able to penetrate well through the cracks along the crystal. Macroscopic crack patterns are often due to different types of microcracks. Therefore, in order to understand the direction of fracture development within the eroded rock mass, further study of the effect of microcracking on macroscopic cracking may be useful.

### Effect of microcracking characteristics on macroscopic cracking

**Fig 15:**
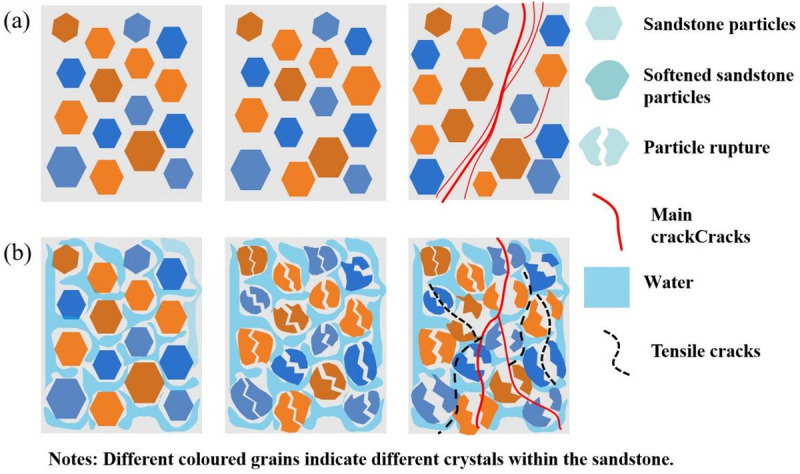
Sandstone cracking mechanism diagram. (a, b denote cracking in uneroded and eroded sandstone, respectively.).

Based on the above findings, it can be concluded that the fractures formed after compression of sandstones undergoing different numbers of freeze-thaw cycles have different characteristics. This phenomenon is related to the characteristics of microcracks. Microcracks induced by water-ice phase transitions during freeze-thaw cycles make the rock matrix discontinuous. Consequently, the higher number of freeze-thaw cycles results in greater rock fragmentation, which is consistent with previous studies [65]. In subsection 4.1 it has been discussed that the types of microcracks are categorised into along-crystal cracks and through-crystal cracks. Thus, the objective of this section is to conduct a comprehensive investigation into the impact of these two types of microcracks on macroscopic cracking and a mechanism diagram for cracking of freeze-thawed sandstone specimens is presented, as shown in Fig 15.

From Fig 15 (a), it can be concluded that the main reason for cracking in the uneroded sandstone is due to the occurrence of slip between particles. Therefore, most of the cracking in sandstone is dominated by shear cracks, and a large number of cracks converge to form a shear zone inside the sandstone. However, when sandstones are subjected to freeze-thaw erosion, intergranular cracking occurs between particles under the action of ice crystal expansion. This intergranular cracking reduces the bonding between particles, so that when the sandstone is subjected to longitudinal pressure, the amount of displacement in the radial direction increases. Therefore, cracking of sandstone after freeze-thaw erosion is mainly dominated by tensile cracking.

In addition, there is a difference in the cracks formed after sandstone cracking before and after freeze-thaw erosion. Sandstones at high freeze-thaw cycle counts form more complex cracks and have greater crack-to-crack connectivity. This is caused by the creation of crystal penetration cracks. As the softening effect of water reduces the stiffness of the particles, the particles are crushed under pressure, resulting in the formation of microcracks, which are perforated cracks. And the formation of perforating cracks is able to penetrate into the intergranular cracks, which results in the formation of flake derived cracks in the sandstone specimens. Thus, the nature of the crystals within the sandstone determines the different cracking patterns.

### Limitations of the theoretical model

In this study, the freeze-thaw process was numerically simulated under the assumptions that heat transfer within pore water is uniform and that interactions between water molecules and mineral particles are negligible. While these assumptions improve computational efficiency, they also introduce certain limitations. For example, in natural sandstone, water migration and ice crystal formation are significantly influenced by capillary forces and local variations in thermal conductivity—factors that are not considered in the current model. Moreover, the omission of water-rock interactions may lead to an underestimation of stress concentration effects induced by ice crystal expansion at pore boundaries, which in turn can affect the initiation and propagation paths of microcracks. Therefore, although the discrete element simulation employed in this study effectively captures the general trends of strength degradation and crack evolution in sandstone under freeze-thaw conditions, further improvements are needed to enhance the accuracy of local stress distribution and fine-scale crack behavior simulation.

## Conclusions

In this study, the effect of freeze-thaw erosion on macroscopic cracking in sandstone is systematically investigated from the perspective of microcracking derivation mechanism. The macroscopic properties of the sandstone and the pattern of change of the acoustic emission signals under freeze-thaw erosion conditions are firstly determined based on the uniaxial compression test, longitudinal wave velocity test and acoustic emission test. The effect of freeze-thaw erosion on discrete metamodel cracking in sandstone is analysed qualitatively and quantitatively. Finally, the mechanism of microcracking influence on macrocracking is discussed.

1. With the maximum number of freeze-thaws (80), the strength of the sandstone specimens decreased by a maximum of 58.3%. The longitudinal wave velocity of sandstone specimens subjected to freeze-thaw erosion produced a significant reduction. The mass of the specimen showed a tendency to increase before decreasing.
2. The evolution of the acoustic emission ringing counts during the initial 60 freeze-thaw cycles is characterised by four distinct phases, which correspond to the four stages of the sandstone compression process. At 80 freeze-thaw cycles, the ringing counts are present in the sandstone at all stages of the compression process, and the trend of cumulative energy is first accelerated and then gradually slowed.
3. A positive correlation between the frequency of fluctuation of the b value and the number of freeze-thaw cycles is present, and its overall tendency is to increase before decreasing. Based on the variation rules of AF and RA, the predominance of shear cracking in sandstone specimens with low freeze-thaw cycles and tensile cracking in sandstone specimens with high freeze-thaw cycles is obtained.
4. The force chain between particles in the model without freeze-thaw cycles is conducted longitudinally and obliquely during compression. The force chain of the model with 80 freeze-thaw cycles is conducted transversely. After high freeze-thaw cycles, the displacement field of the sandstone varies inhomogeneously and produces more tensile cracks.
5. Microdefects are dominated by along-crystal cracks in specimens with lower freeze-thaw numbers and through-crystal cracks at higher freeze-thaw numbers. When damage occurs in uneroded sandstone, the derived crack pattern is relatively uniform. With the intensification of freeze-thaw erosion, the development of cracks gradually becomes complex. And there are many flaky derived cracks generated.
